# Supplementary material for: Hepatocyte Growth Factor-mediated satellite cells niche perturbation promotes development of distinct sarcoma subtypes
Source: eLife. 2016 Mar 17;5:e12116. doi: 10.7554/eLife.12116 (PMC4811764; doi:10.7554/eLife.12116)
Supplement: Supplementary file 1. — DOI: http://dx.doi.org/10.7554/eLife.12116.020 [file elife-12116-supp1.docx]

**Supplementary file 1: list of primers**

**Genotyping**

*Ckm* S 5’-AAGTGATTAACAGCGCATTAGAGC-3’;

*Ckm* AS 5’-TTCAAGGCCGAATAAGAAGGCTGG-3’;

*Hgf* S 5’-ACCTGCAATCCTGGATAACTT-3’;

*Hgf* AS 5’-GGTCCCCAAACTCACCCTGAAGTTCTC-3’;

*Cdkn2a* a1 5'-GTGATCCCTCTACTTTTTCTTCTGACTT-3';

*Cdkn2a* a2 5'-CGGAACGCAAATATCGCAC-3';

*Cdkn2a* a3 5’-GAGACTAGTGAGACGTGCTACTTCCA-3';

*Pax7* 36 5'-TCGTGCTTTACGGTATCGCCGC-3’;

*Pax7* 26 5'-GTGGGGTCTTCATCAACGGTC-3’;

*Pax7* 24 5'-GGGCTTGCTGCCTCCGATAGC-3’.

**Semi-quantitative reverse transcription PCR and real-time PCR**

*Actin* S 5'- AGATCACAGCTCTGGCTCCCA-3';

*Actin* AS 5'-GTGGACAGCGAGGCCAGGATG-3’;

Transgenic *Hgf* S 5’-CAGGACCATGTGAGGGAGAT-3’;

Transgenic *Hgf* AS 5’-TTCCAAGTCGGTTCATCTCTATGTC-3’;

*Alk* S 5’-GCTACTACCGAAAGGGAGGC-3’;

*Alk* AS 5’-GCAACACTCCAAAAGACCATGT-3’;

*Hprt* S 5′-TGACACTGGTAAAACAATGCA-3′;

*Hprt* AS 5′-GGTCCTTTTCACCAGCAAGCT-3′.

**CNV**

*Met* S 5’-CCCAGTTTCTGACTGAGGGA-3′;

*Met* AS 5’-CACCAGAGGAGACCCTTCAC-3′;

*Alk* S 5’-TGACCGACTACAACCCCAAC-3′;

*Alk* AS 5’-GTGTGATGTTTTTCCGTGGCA-3′;

*Kras* S 5’-TCTTGGATATTCTCGACACAGCA-3’;

*Kras* AS 5’-ATACACAAAGAAAGCCCTCCCC-3’;

*Actl6a* S 5’-ACTCTCCAGCTCTCCTGGG-3′;

*Actl6a* AS 5’-CCTAAGGTACAAAGCATAGGCAGT-3′.
